# Response of benthic invertebrate assemblages to seasonal and habitat condition in the Wewe River, Ashanti region (Ghana)

**DOI:** 10.1515/biol-2021-0040

**Published:** 2021-04-08

**Authors:** Samuel K. Oppong, Collins Ayine Nsor, Gabriel Kwabena Buabeng

**Affiliations:** Department of Wildlife and Range Management, Faculty of Renewable Natural Resources, Kwame University of Science and Technology, Kumasi, Ghana; Department Forest, Resources, Kwame Nkrumah University of Science and Technology, Kumasi, Ghana

**Keywords:** benthic invertebrates, stream condition, geometric series, rarefaction, Hill numbers, canonical correspondence analysis, Wewe River

## Abstract

Aquatic macro-invertebrates play a vital role in the food chain of river ecosystem at several trophic guilds and consumer levels, and are used as biomonitoring tools for aquatic ecosystem health. However, hydrologic conditions of these ecosystems have been severely altered because of the increase in urban development and agricultural expansion. This study examined benthic invertebrate response to processes that structure their community in the Wewe River, segmented into intact, medium, and severe condition zones. We sampled in 100 stations in a period of 4 months in the wet (June–September, 2019) and 3 months in the dry (January–March, 2020) seasons. Geometric series, rarefaction, and Hill numbers models were used to quantify invertebrate assemblages, while ordination technique, canonical correspondence analysis, was used to evaluate the influence of predictive factors on their assemblages. A total of 2,075 individuals belonging to 20 family taxa were registered. There was no significant difference in benthic assemblages between the dry and wet seasons. Predictive factors accounted for 47.04 and 50.84% variances, respectively. Taxa distribution patterns differed significantly only in the severely disturbed zone during the wet season. Neptidae, Libellulidae, and Chironomidae were the most abundant taxa, indicating their broad range habitat preference and their ability to adapt to seasonal changes. Asellidae and Perlidae were the least detected, suggesting their sensitivity to elevated levels of some water quality parameters. The findings highlight the threats to the benthic community and overall functional state of the Wewe River, with the need to consider the proposed conservation interventions indicated in this study.

## 1 Introduction

Aquatic macro-invertebrates play an important role in the food chain of an ecosystem at several trophic guilds and consumer levels, and thus reflect ecosystem health [1]. Within the aquatic environment, macro-invertebrates range across a diverse range of microhabitats, with their diversity increasing in areas that provide abundant and diverse resources [2]. Their diversity and abundance are significant community attributes that are controlled by a variety of mechanisms at different spatial scales [3]. These environmental variables, which tend to influence their distribution and abundance, have been documented by a number of studies [3,4]. A good association among macro-invertebrate assemblages [3], which include chemical variables [5,6], organic energy base [7], and habitat-related physical factors such as substrate composition [8], elevation and stream size [9,10], vegetation, geology, and human land use [11,12], and temperature [10,13,14], has been documented as the factors influencing macro-invertebrate community assemblages. Other studies point to hydrologic conditions as the key driving forces affecting distribution and abundance patterns of benthic invertebrates [15,16,17]. For instance, studies on the hydro-climatic trends and variability over the Black Volta in Ghana suggest an increase in warming trends [18], and this phenomenon equally has the potential to impact on benthic invertebrate assemblages.

Changes in benthic invertebrate distributions caused by river regulation may occur because of altered habitat, flow patterns, sediment input, water quality, and thermal regimes [2,6]. Apart from these aforementioned drivers, yearly variations in seasons can be a factor that significantly affect the hydrologic regime and geomorphology in stream environments, by determining the distribution and abundance [12]. For example, a wet season low and a dry season high are expected for periodic seasonal patterns in abundance, depending on the frequency and intensity of summer monsoon rainfall [3].

Streams and rivers in urban centres worldwide have been severely polluted because of increase in urban development [19,20]. In Ghana, rivers draining through urban centres have underwent a significant transformation because of agricultural expansion and infrastructural development [21,22,23,24]. These threats could potentially impact on benthic invertebrate habitat quality, with a probable effect on their abundance, diversity, and spatial distribution. Scientific studies on macrobenthic invertebrates among urban rivers of Ghana remain poor, compared with extensive studies in similar areas in North Africa [25,26,27] and South Africa [28,29]. The few studies in Ghana’s freshwater systems have focused on hydroclimatic trends [18] and safe use of ground water [30]. The Wewe River is one of the few urban systems that drains through patches of the urban forest reserve in the Kumasi Metropolitan Area of Ghana. However, there have been concerns in recent times on the increasing level of human-led disturbances, namely farming activities, sewage disposal, tree felling, and bushfire [24].

Given the lack of scientific information about benthic invertebrates’ status in the Kumasi Metropolitan Area, it is not clear how these disturbance-related drivers have directly influenced physicochemical parameters and the consequent effect on benthic invertebrates. Furthermore, because of their sensitivity to aquatic environments, benthic invertebrates are widely considered as good indicators of water quality [14], by aiding in the identification of anthropogenic disturbances [1]. Thus, understanding how current water physicochemical parameters impact on benthic invertebrate assemblages is critical in choosing the appropriate conservation measures that will help restore the ecological integrity of Wewe River health. Secondly, the study on diversity and distribution patterns of benthic invertebrates and how they are influenced by physicochemical drivers are vital, because these organisms are used to track changes in the biological integrity of ecosystems [2].

In this paper, we assessed seasonal response of benthic invertebrate to physicochemical parameters in the Wewe River. To achieve this broad aim, we sought to answer the following objectives: (1) Are there variations in the abundance and distribution patterns along the three condition zones of the Wewe River? (2) Are there differences in benthic diversity among the three condition zones of the Wewe River? (3) What processes structured benthic assemblages among the three condition zones of the Wewe River? We hypothesized that (a) benthic invertebrate assemblages will differ between seasons, in terms of both taxonomic diversity and number of individuals, because benthic invertebrates have the capacity to recover rapidly from extreme drought periods [31]; (b) physicochemical drivers like dissolved oxygen (DO), total dissolved solids (TDS), electrical conductivity (EC), surface water temperature, salinity, mercury content, water depth, stream flow (slow, medium, and fast), and substrate composition that structure benthic invertebrate communities will vary in the wet and dry seasons, because seasonality tends to directly influence physicochemical drivers.

## 2 Materials and methods

### 2.1 Study area

**Figure 1:**
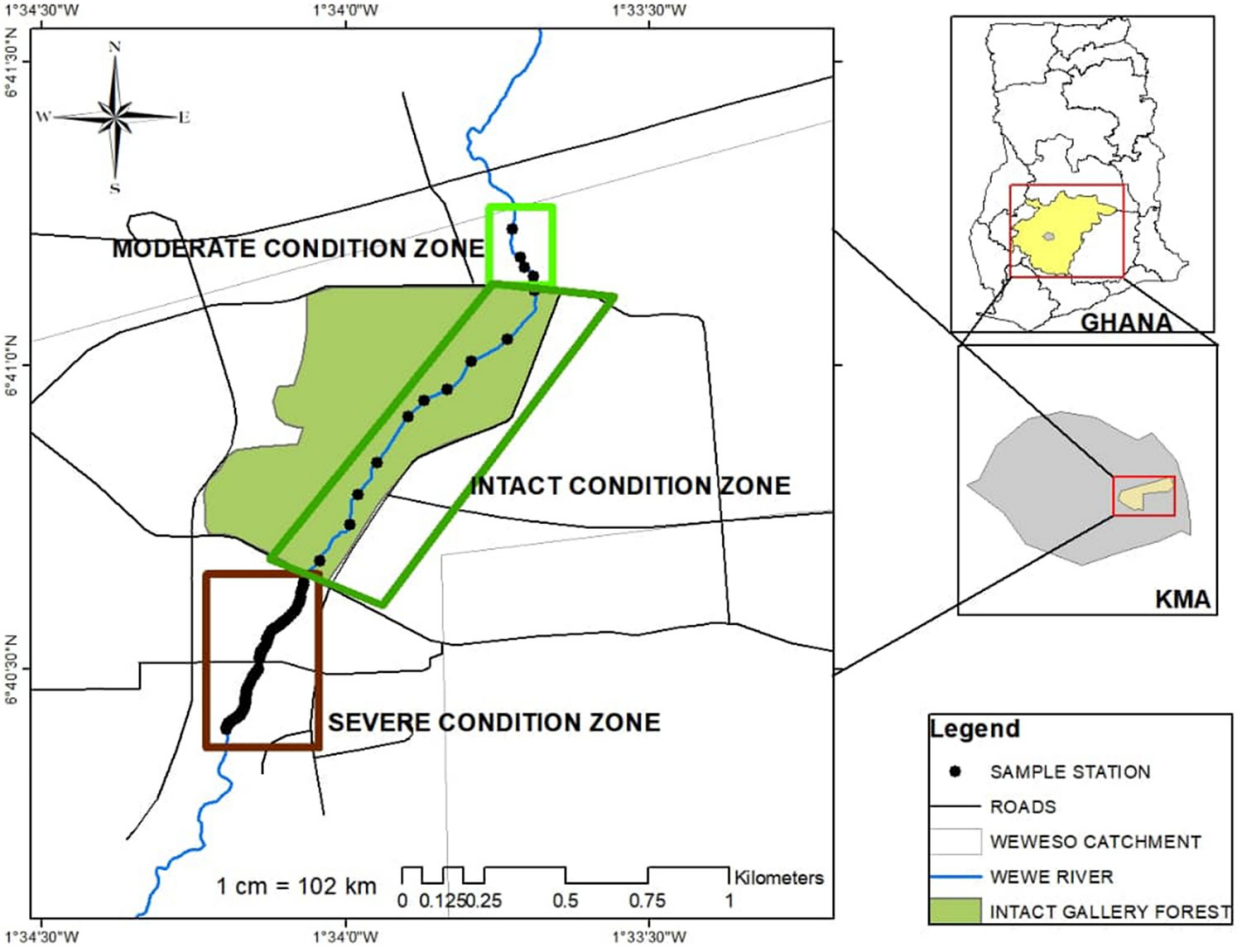
Map of Ghana, showing the study area in the Kumasi Metropolitan Area (Ashanti Region).

**Figure 2:**
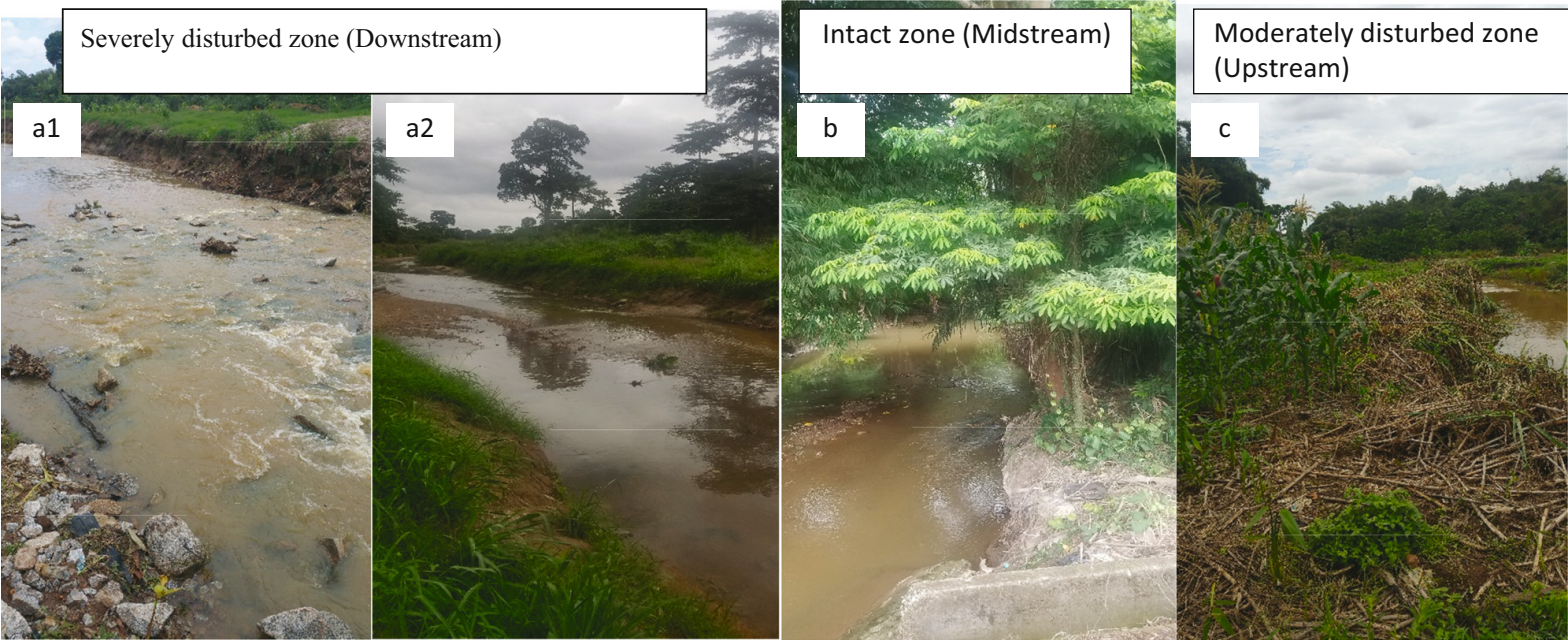
Photographs of some segments of the unregulated Wewe River, where the study was conducted during the wet (June–September, 2019) and dry (January–March, 2020) seasons. a1 & a2 represent the severely disturbed zone (downstream of the river), b = intact zone (river midstream) and c = moderately disturbed zone (river upstream).

The study area is a suburban forest reserve in the Kumasi Metropolis of Ghana and surrounded by communities like Ayigya, Weweso, Bomso, Gyenyasi, Kentikrono, and Ahinsan. Farming along the fringes of the river occurs year round. Waste water from nearby settlements and farm waste equally drains into the river course through direct channel connectivity. Wewe River is a typical unregulated system and takes its source from mountains near Aboabo Nkwanta and flows for about 8.125 km southwest towards Abirem and Weweso [32]. The river is located between N 06°41′30.1″, W 001°33′74.4″ and N 06°40′32.9″, W 001°34′20.9″ (Figures 1 and 2). Soil is typically heavy clay to loamy, characterized by cobbles and boulders. The rock type is igneous and metamorphic rocks, with undulating topography. The average temperature is 24–34°C p.a. and generally humid. Rainfall pattern is typically bimodal, with annual average of 2,000 mm p.a. [33]. The study was conducted in a period of 4 months in the wet (June–September, 2019) and 3 months in the dry (January–March, 2020) seasons, spanning a total of 7 months.

### 2.2 Classification of the sampling sites on the riparian zone

We initially demarcated the upstream, midstream, and downstream of the Wewe River into three condition zones (i.e. intact, moderate, and severe condition zones, following the Riparian Quality Index (RQI) methods). The river course was classified into three condition zones, because it is surrounded by a forest reserve, with some disturbance along the riparian zone, which could have a direct influence on the water quality and consequently on benthic community structure. The index ranges from 1 and 15. Thus, *intact condition class* (10–15): areas dominated by different vegetation strata that cover the full length of the segment, which is linked to natural fluvial forms and slightly fragmented; *moderately disturbed condition class* (7–9): areas with vegetation cover nearly half of the study zone being disturbed; 1–3 m active channel width and about 10–30% exotic and ruderal species present; *severely disturbed condition class* (1–6): areas where 60% of the riparian corridor is reduced by human-led activities; vegetation covering <30% (mainly grasses/herbs and isolated woody species), with channel banks connected to agricultural fields. The end-to-end distance within each condition zone included the following: 180 m (upstream, moderately disturbed condition zone), 937 m (midstream, intact condition zone), and 533 m (downstream, severely disturbed condition zone, with an accuracy of ±5 m) (Figure 1, Plate 1). The RQI methods represent a useful tool for monitoring and evaluating the structure of riparian zones, an element of the river morphological conditions.

### 2.3 Benthic macro-invertebrate sampling procedure

Benthic invertebrates were collected in biweekly intervals for a 7-month period – 4 months in the wet season (June–September, 2019) and 3 months in the dry season (January–March, 2020). There is no rainfall in the dry season, and this reflects in the low flow rate. A total of 100 samples were randomly collected across the three segments (i.e. upstream [moderate condition zone] = 30 samples, midstream [intact condition zone] = 40 samples, and downstream [severe condition zone] = 40 samples), with six replicates per sample point. We sampled each condition zone once per week. Invertebrates were collected using a D-frame sweep net (800 μm mesh size, 690 cm^2^ mouth area, and 1 m length dimensions). The D-frame sweep net was driven deep into the sediment and against the flow direction, to trap all organisms inhabiting the sediment column beneath surface water. The D-frame sweep nets are best used as qualitative or semi-quantitative analyses where a diversity of specimens (e.g. IBI or other indices) is more important than density estimates sampling.

Sediments were collected during low tide to avoid pressure waves displacing the finest surface sediments, and subsequently placed in a petri dish filled with soda water and labelled according to the segment they were sampled. They were subsequently transported to the laboratory, where all organisms were sorted from detritus and inorganic materials by sieving on a mesh hardware cloth basket and stored in 95% ethanol [34]. Organisms were then identified up to the family level with the aid of a stereomicroscope (LEICA MZ6) [35] and taxonomic keys provided by Guide to Freshwater invertebrates [36] and Asian stream guide for identification [37]. Voucher specimens that we could not identify were sent to the Faculty of Biological Sciences for identification.

### 2.4 Measurement of physicochemical parameters

Water quality variables, DO, TDS, EC, surface water temperature, salinity, mercury content (mm Hg), and pH, were measured *in situ* with a multi-probe portable meter (Hanna instrument model H19828). Physicochemical drivers were log transformed where appropriate to achieve normality.

### 2.5 Statistical analysis

**Objective 1:** Are there variations in the abundance and distribution patterns of benthic taxa along the three condition zones of the Wewe River?

#### 2.5.1 Benthic invertebrate abundance distribution along the three condition zones

Both benthic invertebrates and physicochemical drivers’ dataset were initially subjected to square root transformation to homogenize variances and achieve normality [38]. Invertebrate abundance as a measure of diversity was quantified by applying the rank abundance distribution model [39,40]. Geometric series (GS) model was then fitted to the benthic invertebrate data using regression model approach [41], to determine their distribution patterns along the condition zones in the riverine continuum. This model approach was used to test against the null hypothesis (*H*_o_) that invertebrate abundance distribution and richness did not differ in each of the three segments or condition zones classes of the Wewe River.

All registered invertebrate order in each of the three segments of the river were ranked from the most to the least abundant on the rank abundant curve [42], with each species rank plotted on the *x*-axis and the abundance plotted on the *y*-axis. Analysis of covariance was used to test for the significant difference of the equality of the slope of invertebrate abundance distributions among the three segments of the Wewe River.

**Objective 2:** Are there differences in benthic richness and diversity among the three condition zones of the Wewe River?

#### 2.5.2 Analyses of benthic richness and diversity

Individual-based rarefaction model [43] was performed to determine invertebrate richness. The rarefaction curve ({f}_{n})\hspace{.25em}]is defined as follows:

(1) {f}_{n}=E{[}{X}_{n}]=K-{\left(\begin{array}{c}N\\ n\end{array}\right)}^{-1}\mathop{\sum }\limits_{i=1}^{k}\left(\begin{array}{c}N-Ni\\ n\end{array}\right)\ldots ,]

where \hspace{.25em}{X}_{n}] = the number of groups still present in the subsample of “*n*” less than \text{ }K] whenever one group is missing from this subsample, N=\text{total}\hspace{.25em}\text{number}\hspace{.25em}\text{of}\hspace{.25em}\text{items}], K=\text{total}\hspace{.4em}\text{number}\hspace{.4em}\text{of}\hspace{.4em}\text{groups},\hspace{.4em}\text{and}\hspace{.4em}Ni=\text{total}\hspace{.45em}\text{number}\hspace{.45em}\text{of}\hspace{.2em}\text{items}\hspace{.2em}\text{in}\hspace{.25em}\text{group}\hspace{.5em}i\text{ (}i=1,\ldots ,k\text{)}] [42]. Rarefaction methods (both sample and individual-based) allow for a suitable standardization and comparison of datasets with different sampling effort [44] and have been used on invertebrate richness analyses [45,46,47].

Finally, invertebrate diversity was quantified using Hill numbers [48,49,50,51,52]. We used Hill numbers because they incorporate relative abundance and species richness in diversity analysis [49] and defined as follows:

(2) {}^{q}D={\left(\mathop{\sum }\limits_{i=1}^{S}{p}_{i}^{q}\right)}^{1/(1-q)},]

where *S* is the number of species in the assemblage, and the *i*_th_ species has relative abundance *p*_*i*_, *i* = 1, 2,..., *S*.

**Objective 3**: What processes structured benthic assemblages among the three condition zones of the Wewe River?

#### 2.5.3 Analysis of benthic taxa-physicochemical driver relationship

Canonical correspondence analysis (CCA) was used to evaluate the influence of predictive factors on benthic assemblages [53]. CCA is a direct ordination method, with the resulting product being the variability of the physicochemical drivers and benthic invertebrate data [54]. A ridge regression was performed to remove multicollinearity (i.e. perfect correlation with other predictive factors, which tend to inflate variances of the parameter estimates) [55,56]. Mixed ANOVA test (a parametric technique) or split-plot ANOVA was used to test for a significant difference in invertebrate assemblages (abundance, richness, and diversity) across the three condition zones and the physicochemical drivers. Student’s *t*-test was performed to determine seasonal variability among invertebrates’ families and physicochemical drivers. Where significant difference was detected, we further used Tukey’s HSD *post hoc* test to determine the habitats that differed. Spearman’s rank correlation test was performed to evaluate the significant relationship among physicochemical drivers. All the analyses were performed using PAST ver. 3.18 Package [57].

## 3 Results

### 3.1 Seasonal trends in benthic invertebrate composition and individual abundance across the three condition zones

**Figure 3:**
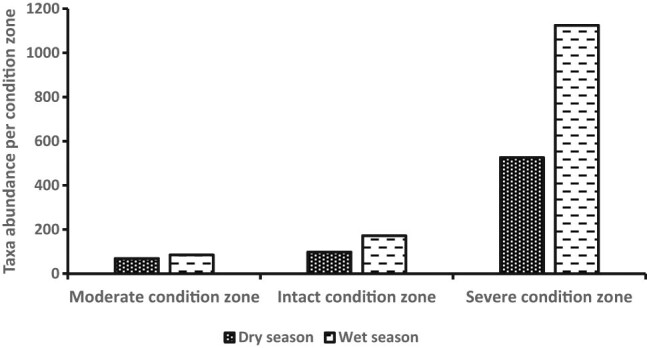
Comparison of seasonal abundance among the three conditions in Wewe River.

**Figure 4:**
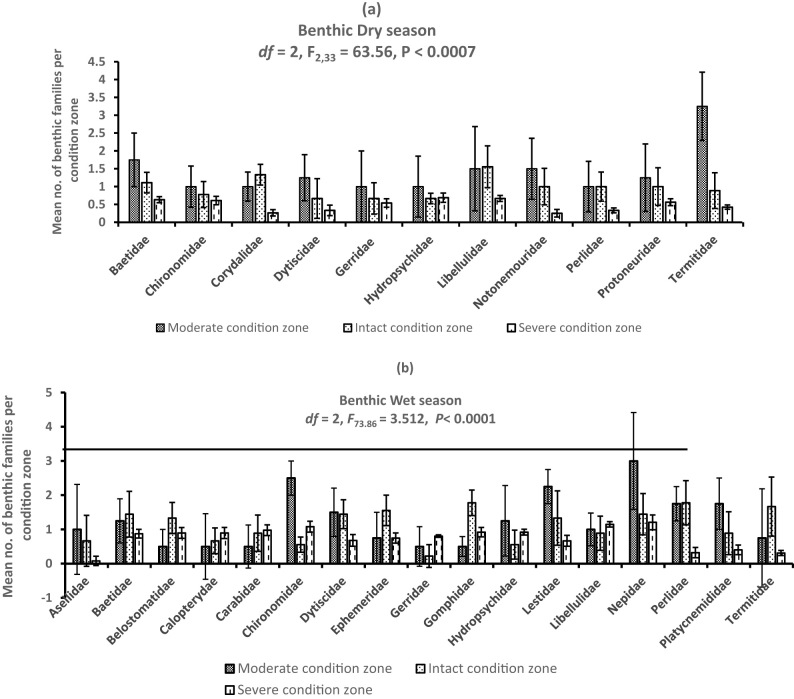
(a and b) Variations in mean composition of benthic families in the Wewe River in the dry and wet seasons. Notice that Chironomidae was the most dominant family across the three habitat condition zones of the river in the wet season.

**Figure 5:**
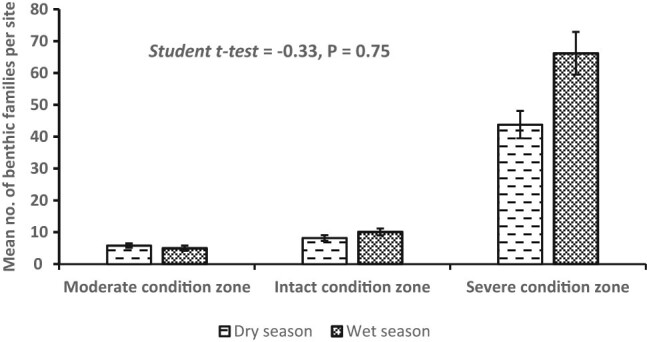
Variations in the seasonal composition of benthic invertebrate along the riverine continuum. Notice that benthic invertebrates were generally higher in the wet season than the dry season. Means are not significantly different.

A total of 2,075 individuals belonging to 20 benthic invertebrate families were registered in the dry (*n* = 693) and wet (*n* = 1,382) seasons and among the habitat condition zones (Figure 3). Variations among mean individuals were substantial in the dry (*F*_2,33_ = 63.56, *P* < 0.0007) and wet (*F*_2,48_ = 73.86, *P <* 0.0001) seasons (Figure 4a and b). Considerable variations among mean number of individuals in the dry season were observed between the moderate and severe (*P* < 0.0003, Tukey’s *post hoc* test) and intact and severe condition zones (*P* < 0.0001), whereas during the wet season, it was between the moderate and severe condition zones (*P* < 0.0007) and intact and severe condition zones (*P* < 0.0006). Although we detected more family taxa in the wet season (*n* = 17) than the dry season (*n* = 12), mean seasonal variations were not substantially significant (Student’s *t*-test = −0.329, *P* = 0.75) (Figure 5). From individual condition zones, the upstream of the river which constitutes the moderate condition zone recorded the highest mean number of individuals per family taxa in the dry (3.25 ± S.E. 0.85) and wet seasons (2.25 ± S.E. 1.3), while the lowest number was registered in the severe condition zone (downstream) during the dry season (0.25 ± S.E. 0.05) and intact condition zone in the wet season (0.22 ± S.E. 0.22) (Figure 4a and b).

### 3.2 Benthic family distribution patterns in the three condition zones

**Figure 6:**
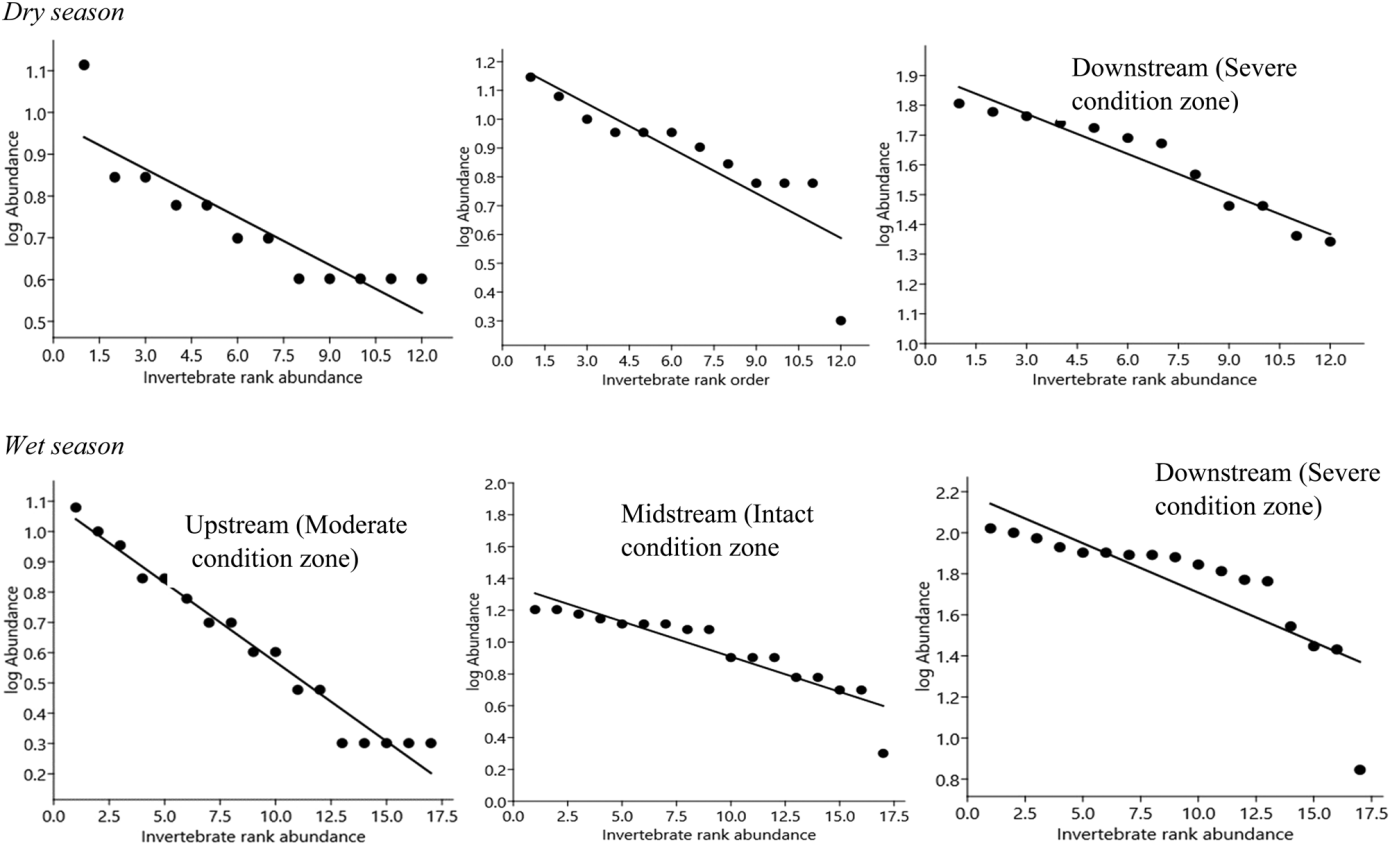
GS model for benthic families rank abundance distribution across the three condition zones in the dry and wet seasons, on the Wewe River. Abundance is based on cumulative count values per sample zone. Notice that benthic families are ordered in decreasing magnitude and plotted against their corresponding rank.

We found in all cases that invertebrate family abundance distribution among the three habitat condition zones fitted well in the GS model (Figure 6). However, comparison between the dry (C.V. = 52.38%) and wet (C.V. = 35.84%) seasons revealed that invertebrates were not significantly distributed (*Z* = −0.517, *P* = 0.61, *Fligner Kileen test for equal* C.V.) (Figure 5). Individual segments of the Wewe River showed that taxa abundance distribution along the curves was not significantly different in the moderate (*χ*²*P* = 2.69, *P* < 0.98), intact (*χ*²*P* = 2.49, *P* < 0.99), and severely disturbed (*χ*²*P* = 5.15, *P* < 0.88) condition zones in the dry season (Figure 6, Table 1). Similarly, in the wet season, there was no substantial difference in the moderate (*P* < 0.901) and intact (*P* < 0.99), with the exception of the severely disturbed condition zone (*χ*²*P* = 75.81, *P* < 0.0004) (Figure 6, Table 1).

**Table 1:** *χ* ^2^ *p* goodness of fit for the GS model for the abundance rank distribution of benthic invertebrate, calculated for all three segments of the Wewe River in the dry and wet seasons. The variable *α* determines the shape of the distribution

River segments	*α*	*χ*²*P*-value for GS	*P*-level
**Dry season**			
Moderate	0.084	2.69	0.98
Intact	0.112	2.49	0.99
Severe	0.098	5.15	0.88
**Wet season**			
Moderate	0.114	0.43	0.90
Intact	0.096	4.76	0.99
Severe	0.105	75.81	0.0004***

Significance of *P*-values: *P < 0.05, **P < 0.01, ***P < 0.0001.

Of the 20 benthic families registered, Libellulidae (*n* = 78), Odontopygidae (*n* = 73), and Baetidae (*n* = 72) were the highest ranked on the abundance distribution curve and were widely distributed across the three condition zones, during the dry season (Figure 6, Table 2). Rarer orders such as Notonemouridae (*n* = 37, 5.3%), Dytiscidae (*n* = 40, 5.7%), and Perlidae (*n* = 42, 6.0%) were the least ranked in the moderate and intact condition zone, reflecting their sensitivity to habitat perturbation. Overall, the severely disturbed condition zone supported the most abundant family taxa.

**Table 2:** Summary of individual benthic invertebrates recorded in the dry and wet seasons, along the three condition zones, in the Wewe River. *N* = 100 sample plots

Macro-invertebrate families	Total number of individuals			Total
	Moderate condition zone	Intact condition zone	Severe condition zone	
**Dry season**				
Baetidae	7	10	55	72
Chironomidae	4	7	53	64
Corydalidae	4	12	23	39
Dytiscidae	5	6	29	40
Gerridae	4	6	47	57
Hydropsychidae	4	6	60	70
Libellulidae	6	14	58	78
Notonemouridae	6	9	22	37
Odontopygidae	7	2	64	73
Perlidae	4	9	29	42
Protoneuridae	5	9	49	63
Termitidae	13	8	37	58
*Total*	69	98	526	693
**Wet season**				
Asellidae	4	6	7	17
Baetidae	5	13	76	94
Belostomatidae	2	12	78	92
Calopterydae	2	6	78	86
Carabidae	2	8	85	95
Chironomidae	10	5	94	109
Dytiscidae	6	13	59	78
Ephemeridae	3	14	65	82
Gerridae	2	2	70	74
Gomphidae	2	16	80	98
Hydropsychidae	5	5	80	90
Lestidae	9	12	58	79
Libellulidae	4	8	100	112
Neptidae	12	13	105	130
Perlidae	7	16	28	51
Platycnemididae	7	8	35	50
Termitidae	3	15	27	45
*Total*	85	172	1,125	1,382

In the wet season, Neptidae (*n* = 130, 9.4%), Libellulidae (*n* = 112, 8.1%), and Chironomidae (*n* = 109, 7.9%) were the highest ranked invertebrate families in all three condition zones, while Asellidae (*n* = 17) and termitidae (*n* = 45) were the least ranked and infrequent taxa, whose distribution occurred in narrow ranges in the three condition zones (Figure 6). These benthic families constituted 1.2 and 3.2%, respectively, of the total number of invertebrates sampled.

Observation from individual condition zones revealed that the abundance and widespread distribution of benthic taxa occurred in the severely disturbed condition zone during the dry and wet seasons, thus reflecting their broad range tolerance to varying physicochemical concentrated levels. Comparison of invertebrate abundant distribution for the three condition zones distinguishes them in relation to the influence of predictive factors. Thus, the shape of the rank abundance curves generally showed differences in invertebrate relative dominance and spatial distribution from individual sample stations.

### 3.3 Benthic invertebrate richness and diversity along the Wewe Riverine system

**Figure 7:**
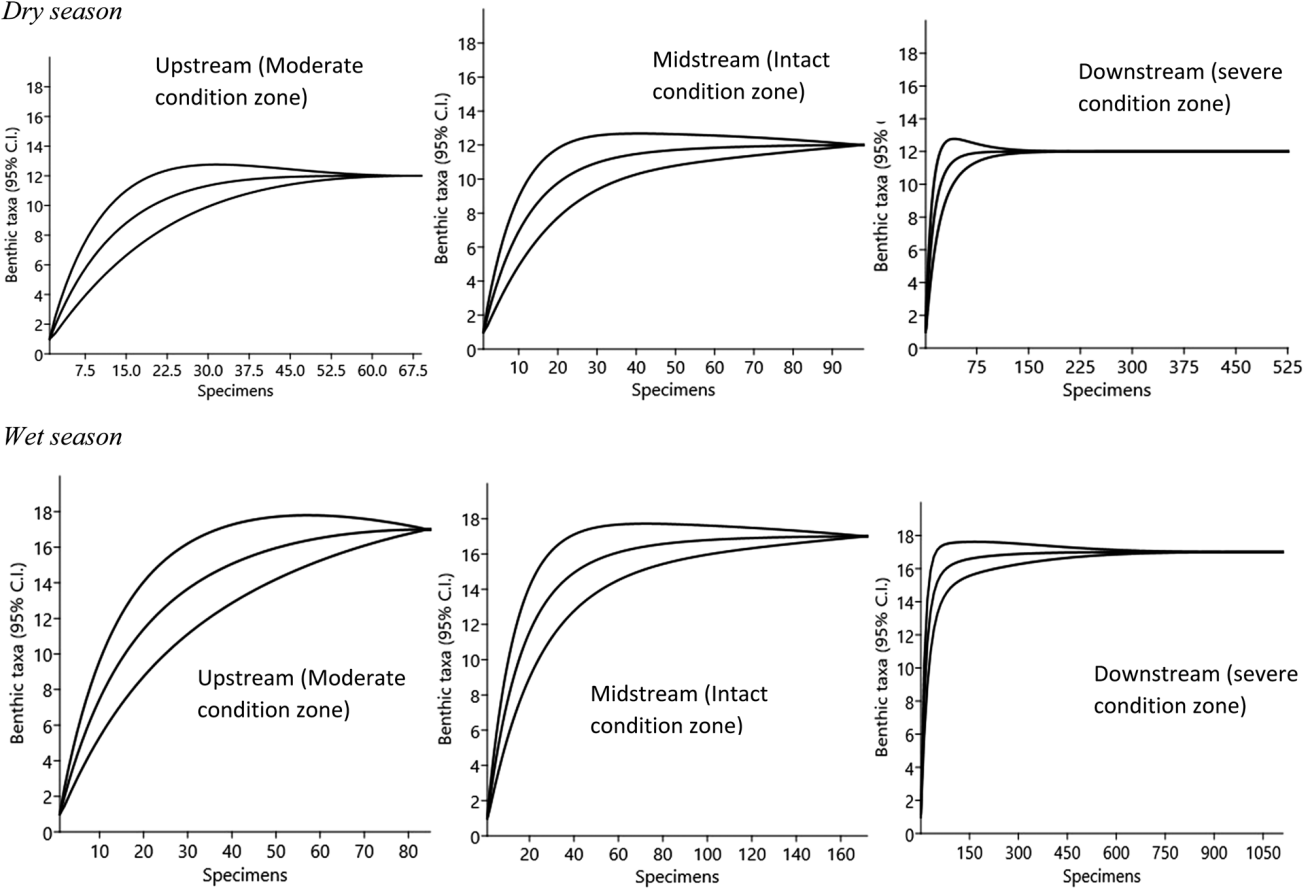
Standardized comparison of benthic invertebrate richness for individual-based rarefaction curves. The data represent summary counts of benthic invertebrates that were recorded from the three condition zones of the Wewe River. The rarefaction curves were calculated from equation (3) (Gotelli and Colwell, 2001), with a 95% confidence interval. The dotted vertical lines illustrate family richness comparison standardized to 69 (dry season) and 85 (wet season) individuals, which was the observed abundance in the upstream of the three segments benthic data set. The average of these individual curves represents the statistical expectation of the species accumulation curve for that particular sample drawn on re-orderings, and the variability among the different orderings is reflected in the specific variance (*conditional*) in the number of families recorded for any given number of individuals.

Seasonal variations in taxa richness were not significant (Student’s *t*-test = 4.335, *P* < 0.62), although the number of benthic families appeared more in the wet season (*n* = 17) than in the dry season (*n* = 12) (Figure 7). Generally, the severe condition zone was the richest in family taxa, while the moderate condition zone was the poorest. Libellulidae was more dominant in the intact (midstream) and severe (downstream) condition zones during the dry season, while Neptidae was dominant in all three condition zones in the wet season (Figure 7). Observed variability in taxa richness and abundance distribution patterns across the three segments of the Wewe River reflected in their diversity profile and ranked from higher to lower diversity indices along the alpha (a) scale values (Figure 7). Condition zone with shallow curve is the most diverse and highest ranked, while those with steep curves are the least diverse and found at the bottom of the Hill number diversity profile.

**Figure 8:**
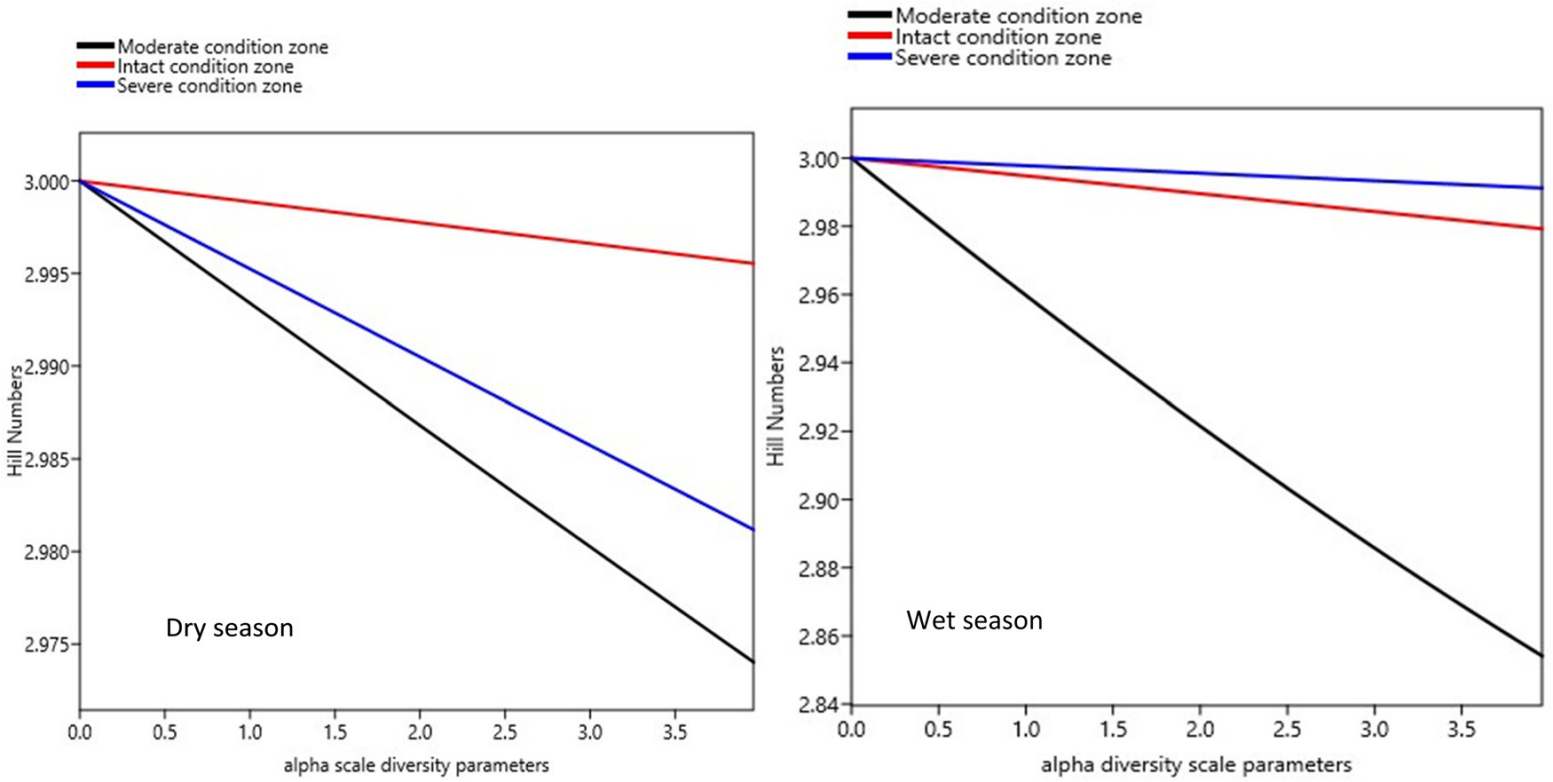
Seasonal trends in benthic invertebrate diversity among the three condition zones, in the Wewe River. Shallower curves reflect high diversity (top of the curves), while steeper curves indicate less diversity (bottom of the curves). Notice that the midstream (intact zone and top red curve) is the most diverse in the dry season, while the downstream (severe condition zone and top blue curve) is the most diverse during the wet season.

Diversity generally did not differ significantly between the dry (*F*_2,6_ = 0.0461, *P* = 0.65) and wet (*F*_2,6_ = 1.06, *P* = 0.40) seasons, and ranged as ^*q*^ *D* = 2.974–2.996 in the dry and wet seasons (Figure 8, Table 3). However, from individual condition zones, we found the intact condition zone (midstream of the river in red colour) (^*q*^ *D* = 2.996) and the severe condition zone (downstream of the river, in blue colour) (^*q*^ *D* = 2.991) to be the most diverse in the dry and wet seasons, respectively (Figure 8, Table 3). Invertebrate diversity appeared similar in the different condition zones, suggesting similar patterns in spatial evenness distribution. The moderate condition zone (upstream of the river) was consistently least diverse in the dry (^*q*^ *D* = 2.974) and wet (^*q*^ *D* = 2.854) seasons. The low diversity in the moderate condition zone appears to reflect in its lowest abundance of individuals in both seasons as well (Figure 8, Table 2).

**Table 3:** Summary of Hill’s number diversity order (at *q* = 0, 1, and 2), along the three condition zones, in the Wewe River. The order *q* is mathematically unified family of diversity indices differing among themselves only by an exponent *q* and indicates their likelihood to include or ignore the relatively rarer species (Hill, 1973). Thus, *q* = 0 represents the number of species in the sample (richness index), *q* = 1 the exponential of the Shannon–Weiner index, and *q* = 2 the reciprocal of the Simpson’s index (i.e. equivalent number of species)

Alpha values	Moderate condition zone	Intact condition zone	Severe condition zone
**Dry season**			
0 (Number of sp.)	12	12	12
1 (exp[Shannon])	10.082	10.627	10.169
2 (Inv.[Gini_Simpson])	9.352	10.286	9.591
Shannon_Evenness	0.916	0.966	0.924
Hill diversity numbers	2.974	2.2996	2.981
**Wet season**			
Alpha values			
0 (Number of sp.)	17	17	17
1 (exp[Shannon])	11.608	12.771	13.249
2 (Inv.[Gini_Simpson)	9.325	12.159	12.775
Shannon_Evenness	0.829	0.912	0.946
Hill diversity numbers	2.854	2.979	2.991

### 3.4 Seasonal environmental influence on benthic community structure across the three condition zones in the Wewe River

**Figure 9:**
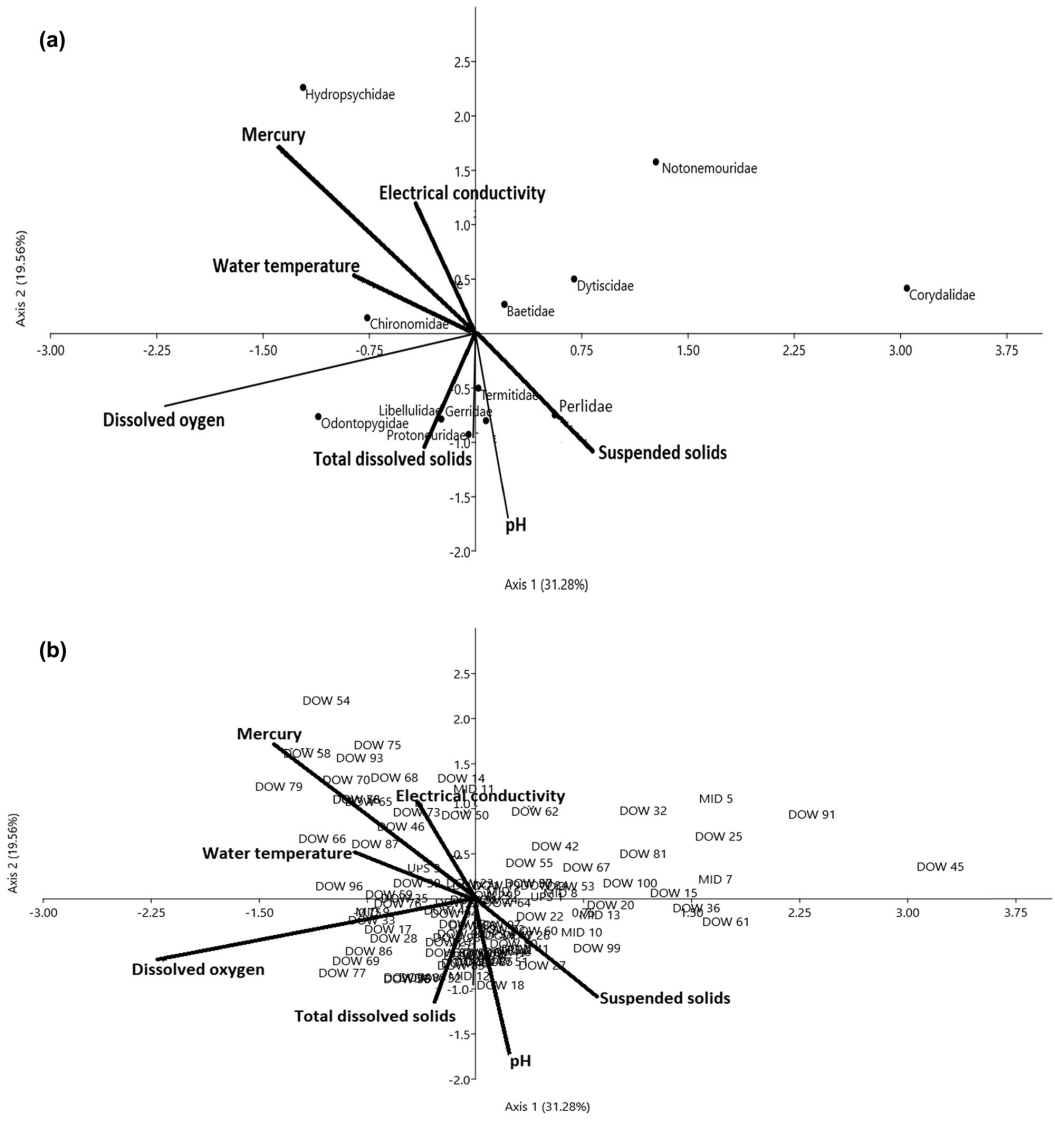
(a and b) CCA diagram showing the influence of physicochemical drivers on benthic invertebrate assemblages in the dry season. The first two axes (Axes I = 31.28 and II = 19.56) explained 50.9% of variations across the three condition zones in the dry season. The arrows represent each of the physicochemical drivers plotted pointing in the direction of maximum change of explanatory variables among the three habitats. Sample plot codes represent each of the three condition zones along the river course (i.e. DOW = downstream of the severe condition zone, MID = midstream of the intact condition zone, and UPS = upstream of the moderate condition zone).

**Figure 10:**
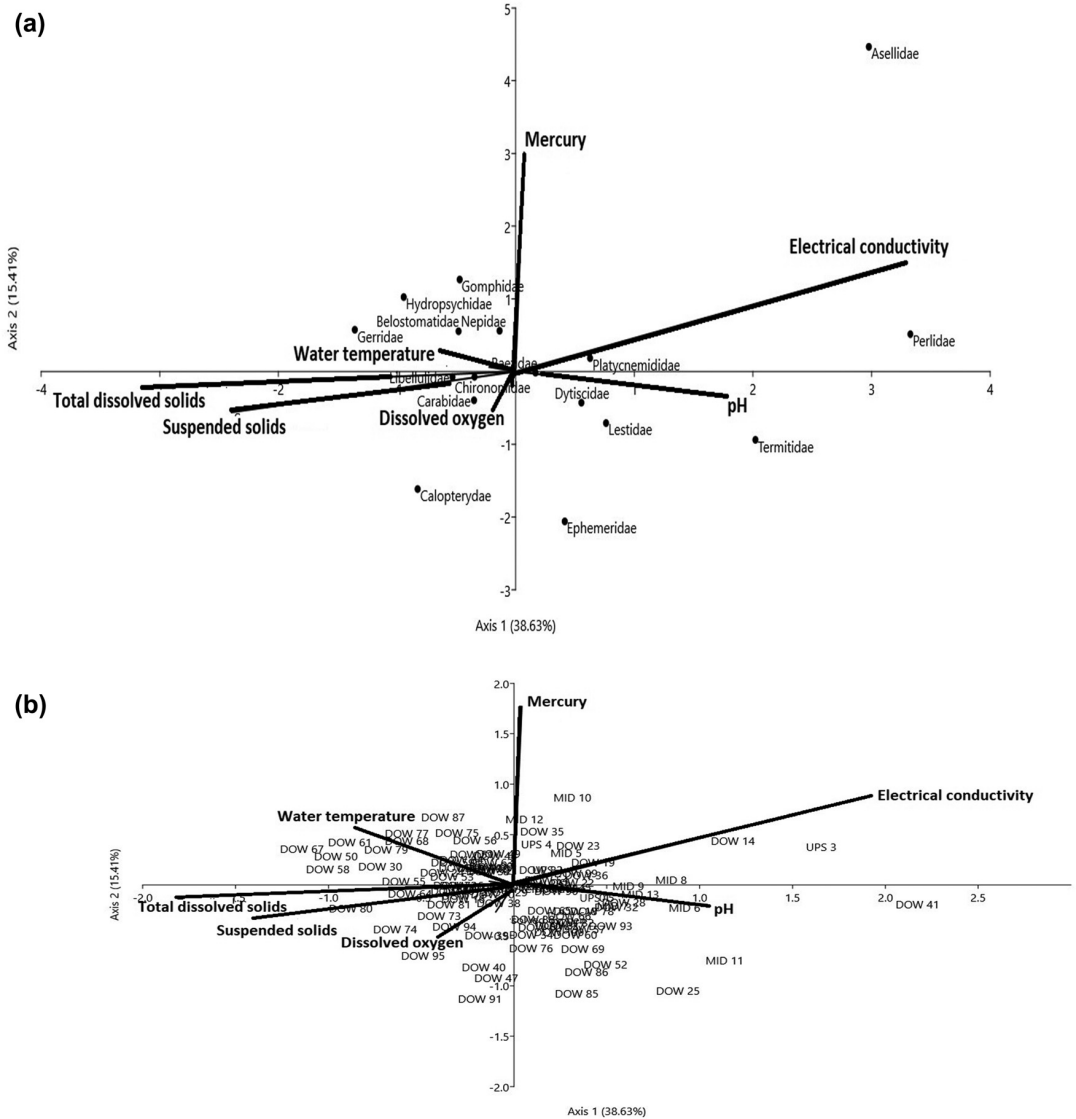
(a and b) CCA diagram showing the influence of physicochemical drivers on benthic invertebrate assemblages in the wet season. The first two axes (Axes I = 31.63 and II = 15.41) explained 47.04% of variations across the three condition zones in the dry season. The arrows represent each of the physicochemical drivers plotted pointing in the direction of maximum change of explanatory variables among the three habitats. Sample plot codes represent each of the three condition zones along the river course (i.e. DOW = downstream of the severe condition zone, MID = midstream of the intact condition zone, and UPS = upstream of the moderate condition zone).

The matrices of the invertebrate-site biplot generated by CCA showed DO (*r* = 0.54, *P* < 0.01) and surface water temperature (*r* = 0.31, *P* < 0.05) on Axis I, and total dissolved solids (*r* = 0.32, *P* < 0.05) and EC (*r* = 0.32, *P* < 0.05) on Axis II, as the major physicochemical drivers of benthic invertebrate assemblages among the three condition zones in the dry season (Figure 9a and b; Tables 4 and 5). Whereas in the wet season, EC (*r* = −0.63, *P* < 0.01)), TDS (*r* = 0.43, *P* < 0.05), and SS (*r* = 0.31, *P* < 0.05) on Axis I and Mercury (*r* = 0.42, *P* < 0.05) and pH (*r* = 0.38, *P* < 0.05) on Axis II were found to influence the community structure of benthic invertebrate (Figure 10; Tables 4 and 5). The first two axes in the dry season (Axes I = 31.28%, II = 19.56%) accounted for 50.84%, while the wet season totaled 47.04% (Axes I = 38.63%, II = 15.41%) of variations in the weighted averages of the 14 invertebrate families in relation to six physicochemical drivers (Table 5). Physicochemical drivers did not differ among the three condition zones of the Wewe River in the dry (*F*_2,18_ = 0.0026, *P* < 0.99) and wet (*F*_2,18_ = 0.044, *P* < 0.95, one-way ANOVA test) seasons. Furthermore, seasonal variability in physicochemical drivers was equally not significant (Student’s *t*-test = −0.033, *P* < 0.97).

**Table 4:** Summary of water quality parameters of the 100 sample stations, across the three condition zones of the Wewe River, in the dry and wet seasons

	Moderate condition zone	Intact condition zone	Severe condition zone
**Dry season**			
Dissolved oxygen (mg/L)	4.6 ± 0.3	3.0 ± 0.2	5.0 ± 0.1
Total dissolved solids (ppm)	88.8 ± 0.6	90 ± 0.5	99 ± 1.2
Electrical conductivity (μS/cm)	329.5 ± 3.8	397.6 ± 4.8	357.6 ± 1.9
Water temperature (°C)	26.4 ± 0.7	27.9 ± 0.3	28.1 ± 0.1
Mercury (mm Hg)	738.1 ± 0.0	738.3 ± 3.3	738.7 ± 0.1
Suspended solids (mg/L)	177.8 ± 1.3	179.7 ± 0.9	185.5 ± 1.04
pH	6.2 ± 0.6	6.0 ± 0.4	5.9 ± 0.1
**Wet season**			
Dissolved oxygen (mg/L)	4.5 ± 0.3	4.9 ± 0.3	5.0 ± 0.1
Total dissolved solids (ppm)	71.3 ± 2.2	57.7 ± 6.4	84.5 ± 1.1
Electrical conductivity (μS/cm)	499.4 ± 17.9	483.4 ± 44.9	169 ± 2.2
Water temperature (°C)	25.9 ± 0.4	26.2 ± 0.2	26 ± 0.1
Mercury (mm Hg)	739.6 ± 0.1	738.8 ± 1.4	739.4 ± 0.1
Suspended solids (mg/L)	199.5 ± 25.6	205.8 ± 8.9	238.3 ± 3.5
pH	6.6 ± 0.3	6.2 ± 0.4	5.3 ± 0.1

**Table 5:** Summary of CCA axis lengths for ground cover, showing the levels of correlation between axes and physicochemical driver gradients, percentage variance of benthic taxa and benthic taxa–physicochemical driver relationship

	Dry season		Wet season	
Axis	I	II	I	II
Canonical eigenvalue	0.184	0.041	0.109	0.035
% variance explained	31.28	19.56	38.63	15.41
No. of variables = 6				
*Correlations*				
DO	0.538*	−0.053	0.033	−0.013
TDS	0.071	−0.317*	0.425*	−0.173
EC	−0.026	0.324*	−0.631**	0.031
Temp.	0.314*	0.145	0.026	0.176
mm Hg	0.013	−0.004	0.133	−0.417*
SS	0.082	−0.109	0.313*	−0.003
pH	0.162	0.076	−0.075	0.377*

In the dry season, Hydropsychidae and Chironomidae correlated positively with optimal levels of surface water temperature (*r* = 0.31, *P* < 0.05) and EC (*r* = 0.32, *P* < 0.05) in the severe condition zone (DOW = downstream), along Axes I and II (upper left of the CCA diagram). On the lower left of the ordination diagram, Libellulidae, Odontopygidae, and Gerridae were negatively associated with total dissolved solids (*r* = −0.32, *P* < 0.05) and DO (*r* = 0.54, *P* < 0.05) on Axes I and II (Figure 9a and b, Table 5). These sections of the river bed were characterized by cobble-gravel, boulders, and a high flow rate. Intercorrelation between DO and suspended solids (SS) (*r*_s_ = 0.76, *P* < 0.01) may be an effect of the high flow rate, causing oxygen to dissolve in the water, and simultaneously carrying floating debris (Figure 9a and b, Table 6). On the lower right of the CCA diagram, we found that weak acidic pH levels appeared to affect the abundance of Perlidae (*n* = 4, 5.7%) and Termitidae (*n* = 8, 11.6%) in the moderate (MID – midstream) and severely disturbed (DOW – downstream) condition zones. The least abundance of Notonemouridae (*n* = 9) and Corydalidae (*n* = 9) in these two zones was largely linked to the influence of EC on Axis II (Figure 9a and b). Taxa found at the middle of the CCA diagram (i.e. Baetidae and Dytiscidae) appeared to exhibit broad tolerance to average levels of all physicochemical drivers assessed in the three condition zones of the Wewe River.

**Table 6:** Summary of Spearman rank (*r*_s_) correlation matrix between the physicochemical drivers across the three habitats on the riparian zone. A correlation above/below ±0.61 is significant at *** *P* = 0.001; ±0.45 at ** *P* = 0.01, and ±0.33 at * *P* = 0.05

	Total dissolved solids	Electrical conductivity	Temperature	Mercury	Suspended solids	pH
**Dry season**						
Dissolved oxygen	0.04	0.135	0.232	0.723***	0.765***	0.854***
Total dissolved solids		0.011	0.595**	0.585**	0.051	0.486**
Electrical conductivity			0.061	0.034	0.746***	0.679**
Temperature				0.026	0.094	0.146
Mercury					0.012	0.006
Suspended solids						0.738***
pH						
**Wet season**						
Dissolved oxygen	0.0003	0.031	0.033	0.848***	0.001	0.090
Total dissolved solids		0.0004	0.005	0.056	0.014	0.344*
Electrical conductivity			0.003	0.087	0.255	0.038
Temperature				0.706***	0.057	0.073
Mercury					0.430*	0.735***
Suspended solids						0.660**
pH						

In the wet season, we observed that increased level of mercury (*r* = −0.42, *P* < 0.05) and EC (*r* = −0.63, *P* < 0.01) on Axes I and II contributed to the low abundance of Asellidae, Perlidae, and Platycnemididae, located in the upper right hand of the ordination diagram. Similarly, the influence of weak to near-neutral pH levels (*r* = −0.38, *P* < 0.05) partly contributed to high abundance of Dytiscidae, Ephemeridae, and Lestidae. However, abundance of Termitidae (*n* = 3) was affected by the near-neutral levels of pH, especially in the moderately disturbed MID – midstream, condition zones (Figure 10a and b, Table 5). The use of organophosphate pesticides to control pest invasion on the nearby vegetable farms and liquid waste discharge from human settlement may have contributed to a decrease in DO concentration (lower left of CCA diagram), and this impacted on Carabidae dominance. This was evidenced in the strong intercorrelation between mercury and DO (*r*_s_ = 0.85, *P* < 0.01) (Figure 10a and b, Table 6). Widespread distribution of Libellulidae (*n* = 112) and Chironomidae (*n* = 109) was found to strongly correlate with total dissolved solids (*r* = 0.43, *P* < 0.05) and SS (*r* = 0.31, *P* < 0.05), on Axis 1. Other taxa, namely Gerridae, Gomphidae, and Hydropsychidae, on the upper left of the CCA diagram, were within the tolerable range of surface water temperature in the severe condition zone (downstream).

## 4 Discussion

### 4.1 Seasonal trends in benthic invertebrate composition and abundance distribution pattern in the Wewe River

Studies of seasonal dynamics in benthic invertebrate communities among smaller lotic systems have been reported by a number of ecologists [58,59,60,61]. Recently, attempts have been made to examine seasonal or short-term temporal variations in invertebrates among a number of large rivers [62]. The findings in this study showed that spatiotemporal changes in water quality in the severe condition might have contributed to highest invertebrate diversity in the severe and intact condition zones, during the wet and dry seasons, respectively, and intact zone during the dry season. For instance, the high amount of DO concentration (4.9–5.0 mg/L) and the optimal water temperature (26.1–28.1°C) in these two zones appeared to favour invertebrate abundance, richness, and evenness distribution (Table 4). Secondly, the presence of rarer taxa (e.g. Chironomidae, Dytiscidae, Gerrida, and Odontopygidae), particularly in the intact zone, probably contributed to highest diversity during the dry season (Table 2). Diversity is considered as a composite index that combines proportional number of individuals, richness, and evenness distribution [49,63] and have been widely used to measure the ecological integrity of ecosystems worldwide. Apart from this composite index for measuring diversity, the concept of species rarity has widely been used to determine a species’ contribution to the diversity (represented by species’ number, abundance, and range area) [64].

Studies in an intermittent river in North Africa found similar variability in diversity of benthic invertebrate community during the dry and wet seasons [25]. However, the high taxa richness in the wet season compared to the dry season contrast the findings of Grohs [61], who reported of higher total richness in macro-invertebrate in summer and fall, compared with winter and spring in the Missouri River. These differences may be because of variations in seasonal habitat conditions in the two biogeographical zones.

Higher taxa abundance in the wet season was thought to coincide with the larval developmental stages and differing responses of taxa to changes in water quality in the dry and wet seasons’ shift, which is largely driven by seasonal-specific disturbances (i.e. intensity of farming practices mostly in the wet season) and changes in the physical environment of the Wewe River. Norris and Thoms [65] argued that some points of the life cycle of benthic invertebrate communities are inextricably linked to biotic and abiotic stream characteristics. However, Ramírez et al. [60] found increase in insect abundance in the dry season compared to the wet season, in tropical low land streams. The authors attributed this to annual temporal changes in stream physicochemistry related to rainfall, with subsequent changes in discharge.

Low abundance of taxa, especially in the moderate and intact condition zones during the dry season, may occur due to the low levels of concentrated DO, related to low water volume and flow rate (Figure 2, Tables 2 and 4). Extreme dryness is a common phenomenon observed in Ghana, during the peak of the dry season (i.e. January–March). This seasonal phenomenon tends to increase evapotranspiration in many rivers and streams including the Wewe River where this study was conducted. Studies in North Africa have found low benthic taxa abundance to be common in many intermittent streams [25,66,67]. These streams are largely characterized by frequent absence of flow and insufficient water levels during peak flows [25,66,67]. These unstable environmental conditions have the tendency to influence water temperature variability and which may in turn affect smaller instars of insects, ionic concentration, and pH [25]. With air temperature projected to increase (2.5°C) over most parts of Africa by the end of the twenty-first century [68], water temperature is likely to increase. This condition could further affect DO levels in most African freshwater systems, including the Wewe River, where this study was conducted. Thus, benthic taxa noted for drought tolerance condition (e.g. Chironomidae and Libellulidae) [69,70,71] may go extinct.

Changes in stream conditions as a result of seasonality and other direct anthropogenic drivers such as farming, dumping of solid waste in streams, and burning can affect some benthic community structure that are sensitive extreme habitat perturbation, and this probably explained why Corydalidae, Notonemouridae, and Protoneuridae were only found during the dry season, whereas Belostomatidae, Gomphidae, and Neptidae thrived only in wet season (Figures 9a and b and 10a and b). The dominance of Nepidae (water scorpion) in the wet season was probably because of their broad range habitat preferences (i.e. ditches, muddy sections of streams, and water area with dead leaves and twigs) (Figure 10a and b). Finally, the presence of the second most dominant taxa like Chironomidae and Libellulidae equally suggests their ability to inhabit or adapt to different hydrologic conditions. Chironomidae and Libellulidae are known to exist in all freshwater wetland types and, are microhabitat selective and play a vital role in food webs [72]. Thus, their large populations are facilitated by the high productivity of freshwater wetlands [72]. Other studies also revealed that Chironomids are also known to be tolerant to disturbances [70,71]. In coastal rivers of southeast Ivory Coast (a neighbouring country on the western border of Ghana), Edia et al. [73] found Chironomidae among the richest taxon diversity in slightly disturbed environment. Thus, Chironomids could be used as an indicator of severe freshwater habitat transformation, giving its resilience to disturbances.

### 4.2 Seasonal physicochemical influence on benthic invertebrate across the three condition zones

Benthic habitats are complex, and a variety of environmental variables acting at multiple spatial scales regulate the composition and distribution patterns of stream macro-invertebrate assemblages in a synergistic fashion [74,75]. For example, latitude, longitude, pH, and stream characteristics like water velocity, width and depth, substrate composition, and concentrations of nutrients and dissolved organic carbon are often important determinants of macro-invertebrate community structure and may also drive patterns in benthic community composition [63,76,77,78,79]. In this study, DO concentration, surface water temperature, TDS, SS, EC, and pH were the physicochemical drivers that influenced higher abundance of benthic invertebrates in the wet season than in the dry season (Figures 9a and b and 10a and b). These physicochemical drivers were probably at optimal levels and within tolerable limit for benthic invertebrates.

In a similar study on seasonal variations in benthic invertebrates, Dudgeon [12] found hydrologic regime and geomorphology in stream environments, as the key determinants of their distribution and abundance. Using multivariate approach, CCA, Jonsson et al. [80] listed pH, water velocity, organic matter, and low canopy openness as the principal predictors of benthic invertebrate community structure in boreal streams. The impact of these predictive factors that cause changes in aquatic environmental conditions may lead to the loss of species, altered community composition, and homogenization of communities [81]. The aftermath of these effects according to Meyer et al. [81] may result in reduced biodiversity and consequently impair the functioning of these habitats [82].

Impact of higher levels of mercury concentration and SS on the low abundance and distribution of Asellidae and Perlidae especially in the moderate condition zone was probably a result of intensive use of agrochemicals on nearby farmlands, deposition of solid waste on the riparian zone, and the flow of liquid waste from surrounding human settlements, during the wet season. An increase in mercury concentration might have contributed to oxygen depletion, which consequently reduced Asellidae population. Such negative impacts on the functional role of invertebrates as litter decomposers could be impaired and consequently have long-term effects on the ecosystem health of the Wewe River, leading to loss of biodiversity. Hellawell [83] revealed that Perlidae and Ephemeridae are among the intolerant groups, and this tends to reflect in their low numbers usually in the agricultural streams. Getwongsa et al. [84] also found Perlidae to decrease in agricultural streams. Thus, with the current rate of farming expansion along the fringes of the Wewe River, there is the likelihood of increase in mercury concentration in the water column (Table 4), through surface run-off during the wet season. Thus, sensitive benthic taxa such as Assellidae and Perlidae [85] could be threatened by this disturbance-related driver. Expansion of cultivated lands along agroecological zones in the humid highlands of Ethiopia was found to have an impact on ecosystem like freshwater systems [86]. This suggests that aquatic life along these agroecological zones may as well be at risk of extinction as a result of these human-led disturbances.

## 5 Conclusion

This study assessed the seasonal response of benthic invertebrate to physicochemical drivers in the Wewe River. Our findings showed that benthic invertebrate assemblages did not differ in the dry and wet seasons. Nonetheless, there was a slight increase among individuals, taxa richness, and diversity in the wet season compared with the dry season. The most severely disturbed segment (downstream) of the Wewe River registered the highest number of individuals, suggesting the role of habitat perturbation and sample size in influencing macro-invertebrate heterogeneity and abundance. Dominance of Chironomidae in the dry and wet seasons was attributed to their broad range habitat preferences and their ability to adapt to seasonal changes in physicochemical driver conditions, while the lowest abundance of Asellidae and Perlidae was probably due to their sensitivity to elevated levels of some water quality parameters, namely mercury and low dissolve oxygen concentration, which were characteristics of the moderately disturbed zone (upstream) of the Wewe River. Thus, for effective management of the Wewe River, we recommend that Asellidae and Perlidae be considered as a suit of indicator benthic orders to monitor the water quality health. Other conservation measures that should be considered in protecting the overall ecosystem of the Wewe River include the following:

(a) farming activities within the riparian zone should be banned and farmers relocated in places further away from the riparian zones;
(b) diversion of liquid sewage spillway emptying into the river course; and
(c) dumping of solid refuse along the riparian zone must be banned by authorities and managers of the Wewe River.

In terms of the setbacks that arose from this study, we noted the difficulty in sampling with less than four persons per segment of the river per day. The extreme stress or tiredness observed among the researchers may have resulted in the lack of detection or loss of some benthic invertebrate samples, as we could not sample every section of the different condition zones of the riverine continuum. We suggest that future studies should involve not less than seven persons, sampling in each segment of a riverine continuum.
